# Validity and reliability of the Checklist for Habitual Physical Activity for people 75 years and older in Japan

**DOI:** 10.1111/psyg.13189

**Published:** 2024-09-09

**Authors:** Kuniko Arita, Yu Ishibashi, Takayuki Tajima, Yoshiaki Ikechi, Hitomi Ishibashi

**Affiliations:** ^1^ Department of Occupational Therapy Graduate School of Human Health Science, Tokyo Metropolitan University Hachioji Japan; ^2^ Department of Physical Therapy, Graduate School of Human Health Sciences Tokyo Metropolitan University Tokyo Japan; ^3^ Kagawa Prefectural Shiratori Hospital Higashikagawa Japan; ^4^ Department of Rehabilitation, School of Health Science Tokyo University of Technology Hachioji Japan

**Keywords:** aged, exercise, human activity, reliability, validity

## Abstract

**Background:**

Despite health benefits, many older adults struggle to meet physical activity guidelines, leading to stagnant activity levels. While integrating physical activity into daily routines has been proposed as a promising strategy for older adults, fit‐for‐purpose measurement tools to assess such routines are lacking. The Checklist for Habitual Physical Activity (CHaPA) was developed based on the positive deviance approach and socio‐ecological model to assess daily behaviours encouraging physical activity among adults aged 75 and older. It has been confirmed for its content and face validity. However, to ensure its broader applicability and usefulness, assessing its construct validity and reliability is necessary. Therefore, this study aims to assess the construct validity and reliability of the CHaPA and accordingly update the tool.

**Methods:**

To validate the construct validity of the CHaPA 22‐item version, we conducted item analysis and exploratory and confirmatory factor analyses. We removed inappropriate items based on predefined criteria. Then, we assessed the reliability, internal consistency, test–retest reliability, and measurement errors of the CHaPA final version.

**Results:**

Item analyses and factor analyses resulted in the deletion of 11 items. The results of confirmatory factor analysis validated the CHaPA 11‐item version with the three‐factor structure based on model fit index with *χ* ^2^/degree of freedom = 1.25, comparative fit index = 0.965, Tucker‐Lewis index = 0.952, and root‐mean‐square error of approximation = 0.038. Omega coefficient (0.90) showed excellent internal consistency. Intraclass correlation coefficient (ICC) demonstrated good test–retest reliability (ICC (1, 2) = 0.77, 95% CI = 0.34–0.89, standard error of measurement = 1.75).

**Conclusions:**

We finalised the CHaPA 11‐item version as a valid and reliable instrument for assessing daily behaviours conducive to physical activity among individuals aged 75 years and older. We need to examine the methods and effectiveness of disseminating this checklist to ensure its utilisation as a resource for promoting healthy ageing and aiding older adults in maintaining active lifestyles.

## INTRODUCTION

Physical activity significantly improves older adults' physical and mental well‐being, potentially extending their healthy life expectancy.^1^ Despite the evident health benefits, many older adults find it difficult to meet current physical activity guidelines, resulting in a stagnant proportion of this demographic meeting recommended activity levels.^2^ Unlike younger adults, who may be robust and active,^3^ individuals aged 75 and older often encounter challenges engaging in physical activity due to declining independence.^4^ As age advances, there is an increased risk of frailty and falls, accompanied by physical limitations due to chronic diseases.^5^ According to a systematic review, global adherence to physical activity guidelines among older adults varies considerably, typically from 2% to 83%, with the majority falling within the 20–60% range.^6^ In Japan, the Ministry of Health Labour and Welfare guidelines, Japan Health 21, emphasises the importance of daily physical activity, stating at least 40 min daily, irrespective of intensity.^7^ The 2019 National Health and Nutrition Survey revealed that only 42.7% of men and 35.9% of women aged 70 years and older engaged in physical activity daily, falling short of the national targets of 58% for older men and 48% for older women.^8^

While integrating physical activity into daily routines has been proposed as a promising strategy for older adults,^9^ more practical tools tailored to individuals aged 75 and older are needed. Guell (2018) emphasised the importance of recognising the significance of daily lived experience, engaging participants in development, and identifying meaningful strategies for physical activity promotion.^10^ Further, despite previous research identifying factors influencing physical activity in older adults, translating these findings into actionable strategies for this demographic remains challenging. For instance, social support has been recognised as a facilitator for physical activity,^11^ and environmental barriers, such as limited access to exercise facilities, have been identified as barriers to participation levels.^12^ To integrate physical activity into daily lives, tools for older adults based on their daily lived experiences, such as how they seek social support and overcome barriers to accessing exercise facilities, are essential.

In response to this need, we developed the Checklist for Habitual Physical Activity (CHaPA), a self‐administered tool specifically designed for adults 75 and older.^13^ The CHaPA aims to assess daily behaviours that facilitate physical activity. Through a qualitative study involving individuals aged 75 and older who were fully physically active, we constructed an item pool for the CHaPA.^14^ The original constructs for the CHaPA were based on the positive deviance (PD) approach^15^ and the socio‐ecological model,^16^ categorising the daily behaviours related to physical activity into the individual, interpersonal, and environmental levels.^17^ The PD approach is an asset‐based, bottom‐up methodology for fostering behavioural and social change within communities. This approach finds solutions to challenges within the community. Despite encountering similar constraints as their peers, individuals identified as ‘positive deviants’ distinguish themselves by identifying and implementing solutions through uncommon or divergent daily behaviours, achieving success.^15^ Older adults aged 75 and older and healthcare professionals confirmed the original CHaPA's content and face validity.^13^ To ensure the tool's broader applicability and utility for people aged 75 and older, assessing its construct validity and reliability within the same population is imperative. Therefore, this study aims to assess the construct validity and reliability of the CHaPA and to establish a final version of the tool.

## METHOD

We conducted a cross‐sectional study utilising self‐administered questionnaires by community‐dwelling older adults in Japan. The study adhered to the Consensus‐based Standards for the Selection of Health Measurement Instruments (COSMIN).^18^

### Participants

We enrolled community‐dwelling older adults aged 75 years and older in the Kanto urban area, Japan. Inclusion criteria were individuals aged 75 years or older who could independently complete the questionnaires twice. Sample size determination followed the guidelines outlined in the COSMIN.^18^ According to these criteria, a sample size equivalent to seven or more times the number of items was considered very good for assessing structural validity. We aimed to recruit a minimum of 154 subjects, which exceeds seven times the number of items in the original version of the CHaPA.

### Recruitment and data collection

We sent written requests to municipalities, senior citizens' associations, and voluntary senior groups via postal mail or email attachments. Upon receiving confirmation from these facilities, we scheduled visits to explain the study to those in charge. Later, we attended group activities at the facilities that have agreed to cooperate for recruitment purposes. Each potential participant received a response set containing a letter requesting research participation, a consent form, a consent withdrawal form, the first and second questionnaires, and a self‐addressed stamped envelope. We distributed the questionnaires between September and November 2023, with submissions collected until December 2023 for analysis inclusion. We formalised the participation through the signing and submission of the consent form.

We instructed participants to complete the first questionnaire and subsequently complete the second questionnaire 7 days later. Both questionnaires were then mailed together to the research facility. Additionally, participants who agreed to provide their name and address for the receipt of an incentive were eligible to receive a gift card valued at 300 Japanese yen (about USD 4.50) via mail.

### Measurements

In the initial questionnaire, participants provided their age and gender. To assess their knowledge regarding national physical activity guidelines,^19^ we asked, ‘Are you familiar with the national physical activity guidelines outlined in Japan Health 21,^7^ as presented by the Japanese government?’ Respondents answered with the following response options: ‘Yes, I am familiar with it,’ ‘I have heard about it, but I am not acquainted with its content,’ or ‘No, I have never heard of it.’

#### The Japanese version of the Physical Activity Scale for the Elderly

The Physical Activity Scale for the Elderly (PASE) evaluates the duration, frequency, intensity, and overall volume of physical activity engaged by individuals aged 65 and older over 7 days.^20^ Following its translation into Japanese,^21^ the Japanese version of the PASE demonstrated satisfactory levels of validity and reliability for utilising the PASE score as an indirect and direct measure of physical activity in Japan.^21^ We contacted the Japanese version's authors to obtain the official questionnaire and a manual.

#### Simple Frail Index

The Simple Frail Index is a self‐administered tool designed to assess the risk of frailty, and its validity has been previously established.^22^ The Simple Frail Index is freely available; no formal permission is required. It comprises five questions about weight loss, walking speed, mobility, memory, and fatigue, each answered with a simple ‘yes’ or ‘no’. Responses are scored accordingly, with a total score of three or more points indicative of frailty, one or two points indicating pre‐frailty, and zero points suggesting robust health.

#### Social frailty index

Social frailty is operationally defined by the presence of specific indicators: ‘living alone,’ ‘reduced frequency of outings compared to the previous year,’ ‘infrequent visits to friends,’ ‘perception of not being helpful to family and friends,’ and ‘lack of daily conversations with others.’^23^ Individuals scoring positively on two or more of these five items indicate socially frail, those scoring positively on one item indicate socially pre‐frail, and those with no positive scores indicate robust health. The Social Frailty Scale is freely accessible for public use and does not require formal permission.

#### SARC‐F

The SARC‐F is a widely utilised screening tool for sarcopenia, with a validated Japanese version available.^24^, ^25^ The SARC‐F comprises five questions assessing strength (S), assistance with walking (A), rising from a chair (R), climbing stairs (C), and falls (F). Each item is rated on a scale of 0–2, ranging from ‘not at all’ to ‘very difficult.’ The total score ranges 0–10. A cutoff score of four points or higher identifies individuals at risk for sarcopenia.^25^

#### Checklist for Habitual Physical Activity

CHaPA, a self‐administered screening tool comprising 22 items, was designed to evaluate the daily behaviours conducive to physical activity among individuals aged 75 and older. Previous studies have confirmed the tool's face and content validity.^13^ The total score is derived by assigning one point for each ‘yes’ response and zero points for each ‘no’ response, resulting in a score ranging from zero to 22.

The second questionnaire asked for the response date, age, gender, and the CHaPA.

### Ethical consideration

Participants were informed about the research objectives and procedures and asked to provide written consent before joining the study. They were assured of the voluntary nature of their participation and their right to withdraw without consequences. The study was approved by the Research Ethics Committee of the Graduate School of Tokyo Metropolitan University (approval number: 23023).

### Statistical analyses and criteria

Descriptive statistics were employed to characterise the sample. The Shapiro–Wilk test was applied to assess the normality of response distribution of the CHaPA 22‐item version. We initially conducted item analyses to identify and remove inappropriate items,^26^ followed by an exploratory factor analysis (EFA) to determine the number of factors present. Subsequently, we conducted confirmatory factor analysis (CFA) to evaluate the models' fit. The significance level was set at 5% for all analyses. We performed data analyses using the RStudio program (version 2023.12.1 + 402).

#### Item analyses

We calculated endorsement frequencies and conducted statistical tests to examine potential gender and age group differences using the Wilcoxon and Kruskal‐Wallis rank‐sum tests, respectively. Additionally, we assessed inter‐item correlations, item‐total (I‐T) correlations, and good‐poor (GP) analyses for each item.^26^, ^27^ We excluded inappropriate items based on criteria: low endorsement frequency and significant gender or age differences.^28^ To maintain the integrity of the checklist as a health promotion tool, an upper limit for the endorsement rate was not established as an exclusion criterion. We used Spearman's rank correlation analysis to examine inter‐item and I‐T correlations. The removal criterion for inter‐item correlation was set at *r* ≥ 0.80.^29^ In contrast, the removal criterion of the I‐T correlation was defined as less than 0.20.^30^ G‐P analysis compared each item using the Mann–Whitney *U*‐test between two groups, the upper and lower 25% of total CHaPA scores.^26^

#### Factor analyses for construct validity

After removing irrelevant items based on the results from item analyses, we assessed the adequacy of the sample for factor analysis using the Kaiser‐Mayer‐Olkin (KMO) test. KMO values should be equal to or greater than 0.70 for factor analysis to be considered appropriate.^31^ We conducted EFA using robust weighted least squares with oblimin rotation to determine the underlying factor structure.^32^ The number of factors was determined using techniques such as minimum average partial (MAP) and parallel analysis. Items with factor loadings below 0.30 were considered inadequate, and those exhibiting cross‐loadings or failing to load uniquely on individual factors were potentially removed.^33^ We named the factors based on the results of the EFA.

We subsequently performed CFA to assess the fit of the model. Goodness‐of‐fit was evaluated based on the following indices: the normed Chi‐square / degree of freedom (*χ* ^2^/df) < 5.00, root‐mean‐square error of approximation (RMSEA) < 0.08, comparative fit index (CFI) ≥ 0.90, and Tucker‐Lewis index (TLI) ≥ 0.90.^34^

#### Criterion‐related validity

We conducted Spearman correlation tests to assess the criterion‐related validity of the CHaPA. The construct being evaluated, namely ‘behaviours that promote habitual physical activity,’ was presumed to be correlated with the amount of physical activity. Previous findings have indicated a weak association between the amount of physical activity and guidelines recognition.^19^ Therefore, external criteria for the hypothetical scale were established, including a strong positive correlation with the amount of physical activity, frailty status, and sarcopenia status. Conversely, we hypothesised a weak positive correlation between knowledge about national physical activity guidelines and the total score of the CHaPA. Correlation coefficients between 0.70 and 1.0 indicate a strong association, between 0.40 and 0.60 a moderate one, and between 0.10 and 0.30 a weak association.^35^

#### Reliability

We assessed internal consistency using the omega coefficient, with a value of 0.70 or higher, which was typically considered acceptable.^36^ Test–retest reliability was evaluated through intraclass correlation coefficient (ICC) analysis, employing a 1‐week interval with the same participants to ensure stability. ICC values less than 0.50, 0.50–0.75, 0.75–0.90, and greater than 0.90 indicate poor, moderate, good, and excellent reliability, respectively.^37^ We estimated measurement error by computing the standard error of measurement (SEM), which was the square root of the participants' within‐subject variance. We also calculated the smallest detectable change (SDC).^38^

## RESULTS

**Figure 1:**
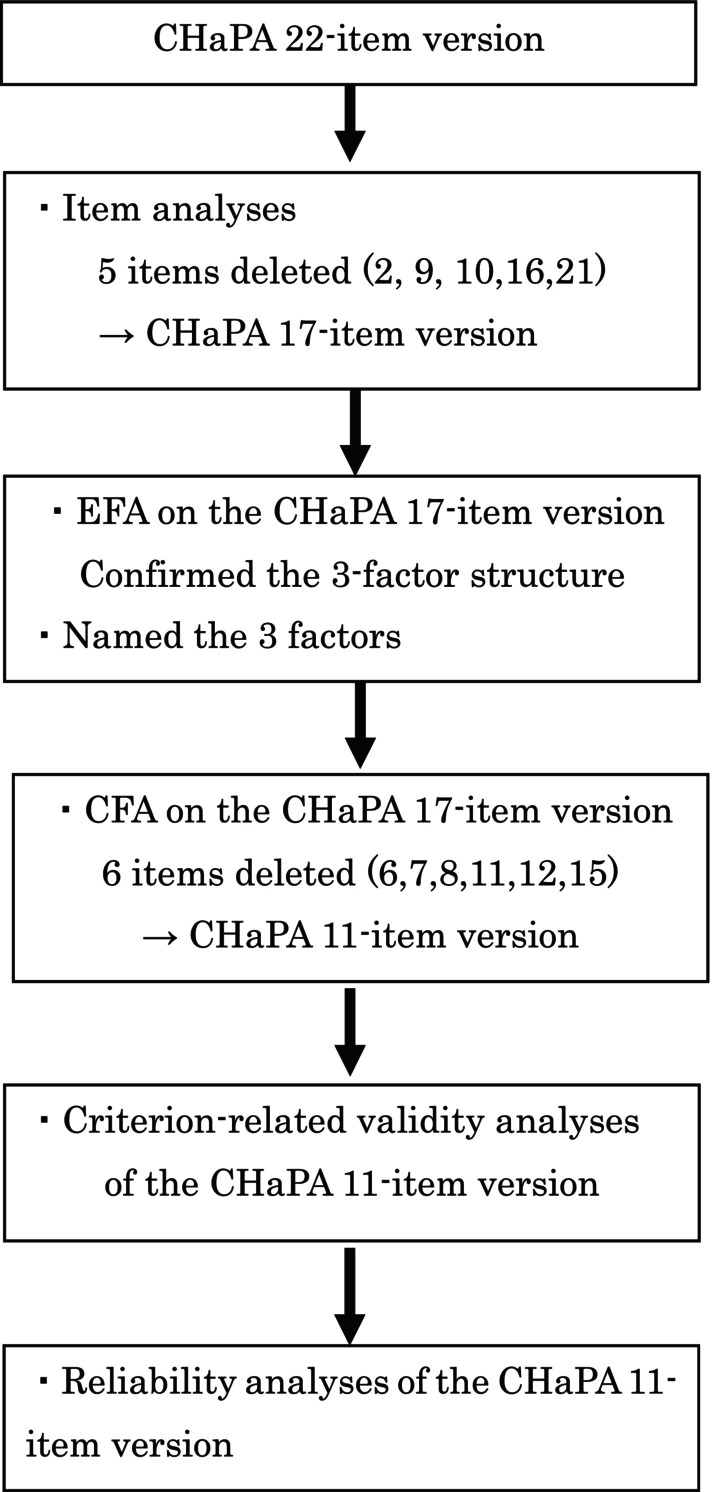
Flow diagram of item deletion and validity, reliability testing process. CHaPA, Checklist for Habitual Physical Activity, CFA, confirmatory factor analysis; EFA, exploratory factor analysis

In collaboration with a health promotion officer from City A, two elderly welfare supporters from City B, and one elderly welfare supporter from District C, four individuals participated as research collaborators, setting up an opportunity to distribute the questionnaires. Of the 339 questionnaires distributed, 181 responses were received, yielding a response rate of 53.4%. Subsequently, 10 response datasets with missing values for the first CHaPA and PASE assessment were excluded, resulting in 171 datasets for analysis. Figure 1 illustrates the sequential process of item deletions, validity, and reliability analyses.

### Characteristics of the participants

Table 1 shows the characteristics of the participants (*n* = 171) with a mean age of 81.02 years (SD = 4.37, range: 75–95). About 77% were female, and 23% were male. Regarding knowledge about the national physical activity guidelines, no one reported familiarity with the guidelines, while 28% indicated awareness, and 72% had never heard of the guidelines, respectively. Seventy percent of participants lived with someone, whereas 30% lived alone. Regarding frailty status, about 27% were classified as robust, 59% were in a pre‐frailty state, and 14% were classified as frailty status. Social frailty analysis revealed that about 31% were robust, 42% were in a pre‐social frailty state, and 27% were in a social frailty status. Additionally, about 15% of participants were identified as having sarcopenia, while 85% did not report signs of sarcopenia. The distribution of the CHaPA total score exhibited non‐normality, skewed toward high scores, and displayed flatter characteristics (*W* = 0.96, *P* > 0.01, skewness: −0.47, kurtosis: −0.39). The mean score was 16.61, with a SD of 3.71, indicating the absence of ceiling and floor effects.

**Table 1:** Basic characteristics of the study participants

Characteristics		Construct validity (*n* = 171)	Test–retest reliability (*n* = 165)
		*n* (%)	*n* (%)
Age (years)	Min	75.00	75.00
	Max	95.00	95.00
	Median	80.00	80.00
	Mean ± SD	81.02 ± 4.37	81.02 ± 4.43
Gender	Female	132 (77.19)	126 (76.36)
	Male	39 (22.81)	39 (23.63)
Knowledge about the national PA guideline	Familiar with it	0 (0.00)	0 (0.00)
	Hard of it, but not familiar with it	47 (27.65)	45 (27.43)
	Never heard of it	123 (72.35)	119 (72.56)
Living alone	Living alone	52 (30.41)	48 (29.09)
	Living with someone	119 (69.60)	117 (70.09)
Simple Frail Index	Robust	46 (26.90)	46 (27.79)
	Pre‐frail	101 (59.06)	95 (57.57)
	Frail	24 (14.04)	24 (14.54)
Social Frailty Index	Robust	53 (30.99)	53 (32.12)
	Pre‐social frail	72 (42.11)	68 (41.12)
	Social frail	46 (26.90)	44 (26.66)
SARC‐F	No	145 (84.79)	139 (84.24)
	Yes	26 (15.20)	26 (15.75)
PASE	Min	0.00	0.00
	Max	329.16	329.16
	Median	111.00	110.90
	Mean ± SD	120.35 ± 55.35	119.30 ± 54.70

PA, physical activity; PASE, Japanese version of the physical activity scale for the elderly; SARC‐F, strength, assistance walking, rising from a chair, climbing stairs, and falls.

### Validity

#### Item analysis

Table 2 presents the outcomes of the item analyses. Per the removal criteria outlined in the method section, we eliminated Items 2, 9, 10, 16, and 21. No significant gender differences were observed across all items. However, significant response variations were noted among the three age groups (70s, 80s, and 90s) for Items 2 and 16. Inter‐item correlation analyses revealed correlation coefficients ranging from −0.13 to 0.53 for each item, indicating no need for item deletion.^29^ Table S1 provides detailed results of the inter‐item correlation analyses. In terms of I‐T correlation analyses, Items 2, 9, 10, and 21 were deemed for deletion due to correlation coefficients falling below 0.20.^30^ Additionally, G‐P analysis indicated non‐significant differences between the upper and lower 25% groups for Items 9, 10, and 21, rendering them eligible for deletion.

**Table 2:** Item analysis of the CHaPA 22‐item version

Item	Endorsement frequency	Gender difference		Age group difference^a^		Inter‐item correlation	I‐T correlation	G‐P analysis	
	*n* (%)	χ^2^	*P*	χ^2^	*P*	*r* range	r	χ^2^	*P*
1: Timeframe for physical activity is determined on a daily, weekly, or monthly basis	140 (81.87)	1.32	0.25	0.23	0.86	−0.05 to 0.34	0.47	24.98	<0.01**
2: Waking up at a set time	158 (92.40)	0.14	0.71	8.99	0.01*	−0.09 to 0.21	0.14	3.91	0.048*
3: Maintaining a habit of walking to the store	123 (71.93)	2.08	0.15	0.19	0.91	−0.08 to 0.28	0.31	19.09	<0.01**
4: Using units that are easy to understand (metres, minutes, steps) to keep track of activity level	114 (66.67)	0.34	0.56	2.12	0.34	−0.02 to 0.34	0.58	39.57	<0.01**
5: Tracking activity needed when travelling to neighbourhood landmarks (e.g., bus stops, supermarkets)	134 (78.36)	0.83	0.36	1.34	0.51	−0.04 to 0.53	0.44	23.41	<0.01**
6: Engaging in physical activity to the point of feeling tired	137 (80.12)	0.64	0.43	1.16	0.56	−0.06 to 0.38	0.41	21.88	<0.01**
7: Regularly measuring values related to your physical status (e.g., weight, blood pressure, body fat)	145 (84.80)	1.00	1.00	2.26	0.32	−0.08 to 0.19	0.24	5.74	0.016*
8: Being aware of which parts of the body are affected by your physical activity	115 (67.25)	0.01	0.92	0.61	0.74	−0.06 to 0.35	0.43	26.99	<0.01**
9: Completing the parts you can do, regardless of the level of accomplishment	162 (94.74)	0.56	0.75	0.77	0.68	−0.09 to 0.22	0.1	0.045	0.83
10: Doing physical activity according to your standards, even if different from the national guidelines	155 (90.64)	1.34	0.25	2.34	0.31	−0.13 to 0.19	0.1	0.28	0.59
11: Incorporating movements from others and media information related to physical activity	106 (61.99)	3.08	0.08	0.82	0.66	−0.04 to 0.35	0.58	40.54	<0.01**
12: Doing familiar physical activities (e.g., things you did when you were young, when you were a child)	93 (54.39)	0.22	0.64	0.82	0.66	−0.07 to 0.30	0.49	37.42	<0.01**
13: Getting daily enjoyment out of physical activity (e.g., meeting people, getting close to nature, observing the environment)	150 (87.72)	2.27	0.13	2.38	0.3	−0.04 to 0.43	0.39	13.88	<0.01**
14: Having a purpose other than exercise (e.g., looking after the community, visiting friends) for your physical activity	127 (74.27)	0.41	0.52	1.87	0.39	−0.07 to 0.43	0.52	29.83	< 0.01**
15: Talking to others during physical activity	163 (95.32)	2.09	0.15	0.55	0.76	0.08–0.40	0.27	3.91	0.048*
16: Doing physical activity in the presence of people older than yourself	121 (70.76)	2.70	0.10	28.05	<0.01**	−0.01 to 0.33	0.49	28.73	<0.01**
17: Having a group role when doing physical activity with others (e.g., preparing and cleaning up, taking care of people and pets, managing equipment)	114 (66.67)	0.04	0.85	1.61	0.45	−0.06 to 0.52	0.5	35.13	<0.01**
18: Talking to family and friends about the physical activity you are doing	149 (87.13)	1.83	0.18	0.55	0.76	−0.07 to 0.33	0.49	21.97	<0.01**
19: Talking to your family doctor about the physical activity you are doing	110 (64.33)	0.36	0.55	0.78	0.68	−0.11 to 0.35	0.45	22.97	<0.01**
20: Setting walking courses taking into account the local environment (e.g., crime prevention, safety, restrooms, resting places)	94 (54.97)	0.00	1.00	1.89	0.39	−0.13 to 0.33	0.53	35.47	<0.01**
21: Using public transportation (e.g., buses) in combination when you are tired or have luggage	146 (85.38)	9.11	1.00	1.63	0.44	−0.06 to 0.24	0.18	1.29	0.25
22: Regularly utilising nearby stairs, slopes for physical activity	95 (55.56)	1.35	0.25	0.66	0.72	−0.06 to 0.27	0.44	38.4	<0.01**

^a^ Age groups were divided into three groups: 75–79, 80–89, and 90–95.

CHaPA, Checklist for Habitual Physical Activity; G‐P, good‐poor; I‐T, item‐total.

* *P* < 0.05;

** *P* < 0.01.

#### Factor analysis

##### EFA

**Figure 2:**
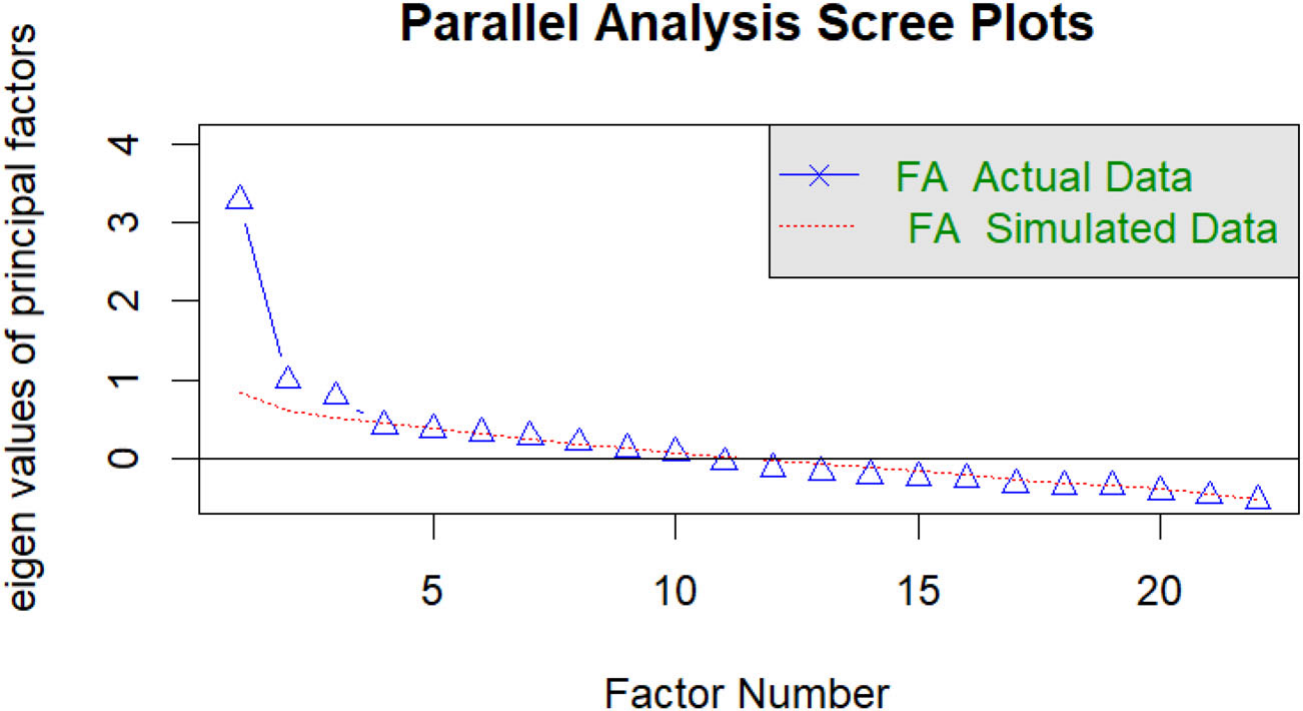
Scree plot. FA, factor analysis

A KMO value of 0.75 indicated the sample's adequacy for factor analysis. Examination through parallel analysis and scree plot (Fig. 2) suggested a three‐factor structure based on the MAP criterion, while the Bayesian Information Criterion (BIC) suggested a two‐factor structure. Subsequently, EFA was performed using robust weighted least squares with oblimin rotation to ascertain the underlying factor structure. Table 3 displays the factor loadings of the three‐factor structures.

**Table 3:** Factor loadings of three‐factor structure (weighted least squares, oblimin rotation)

Item number	Factor loadings			Uniqueness
	Factor 1	Factor 2	Factor 3	
CHaPA4	0.639	−0.034	0.169	0.492
CHaPA5	0.547	−0.065	0.117	0.654
CHaPA22	0.517	0.072	−0.14	0.75
CHaPA3	0.467	−0.151	−0.103	0.813
CHaPA8	0.424	0.113	−0.02	0.792
CHaPA20	0.413	0.131	0.041	0.769
CHaPA12	0.401	0.226	−0.09	0.773
CHaPA14	−0.026	0.841	−0.005	0.304
CHaPA17	−0.007	0.596	0.057	0.63
CHaPA13	0.111	0.470	−0.101	0.759
CHaPA11	0.307	0.389	0.055	0.671
CHaPA7	−0.012	0.161	0.136	0.949
CHaPA1	0.057	−0.062	0.789	0.357
CHaPA15	−0.152	0.195	0.437	0.778
CHaPA19	0.028	0.156	0.416	0.764
CHaPA18	0.096	0.293	0.359	0.693
CHaPA6	0.109	0.18	0.304	0.806
Sums of squares of loadings	1.956	1.878	1.413	
Factor correlation				
Factor 1	1	0.241	0.385	
Factor 2	0.241	1	0.205	
Factor 3	0.385	0.205	1	

CHaPA, Checklist for Habitual Physical Activity.

We labelled Factor 1 as ‘utilising one's living area resources’ and comprised Items 3, 4, 5, 20, and 22. These items pertained to the utilisation of environmental features. We labelled Factor 2, Items 13, 14, and 17, as ‘integrating one's enjoyment, purpose, and roles.’ This factor was associated with integrating physical activity with enjoyable, purposeful, and role‐oriented activities. We labelled Factor 3, Items 1,18, and 19, as ‘planning physical activity and sharing it with others.’ This factor was associated with organising physical activity plans, seeking social support from family and friends, and consulting with primary care physicians.

##### CFA

**Figure 3:**
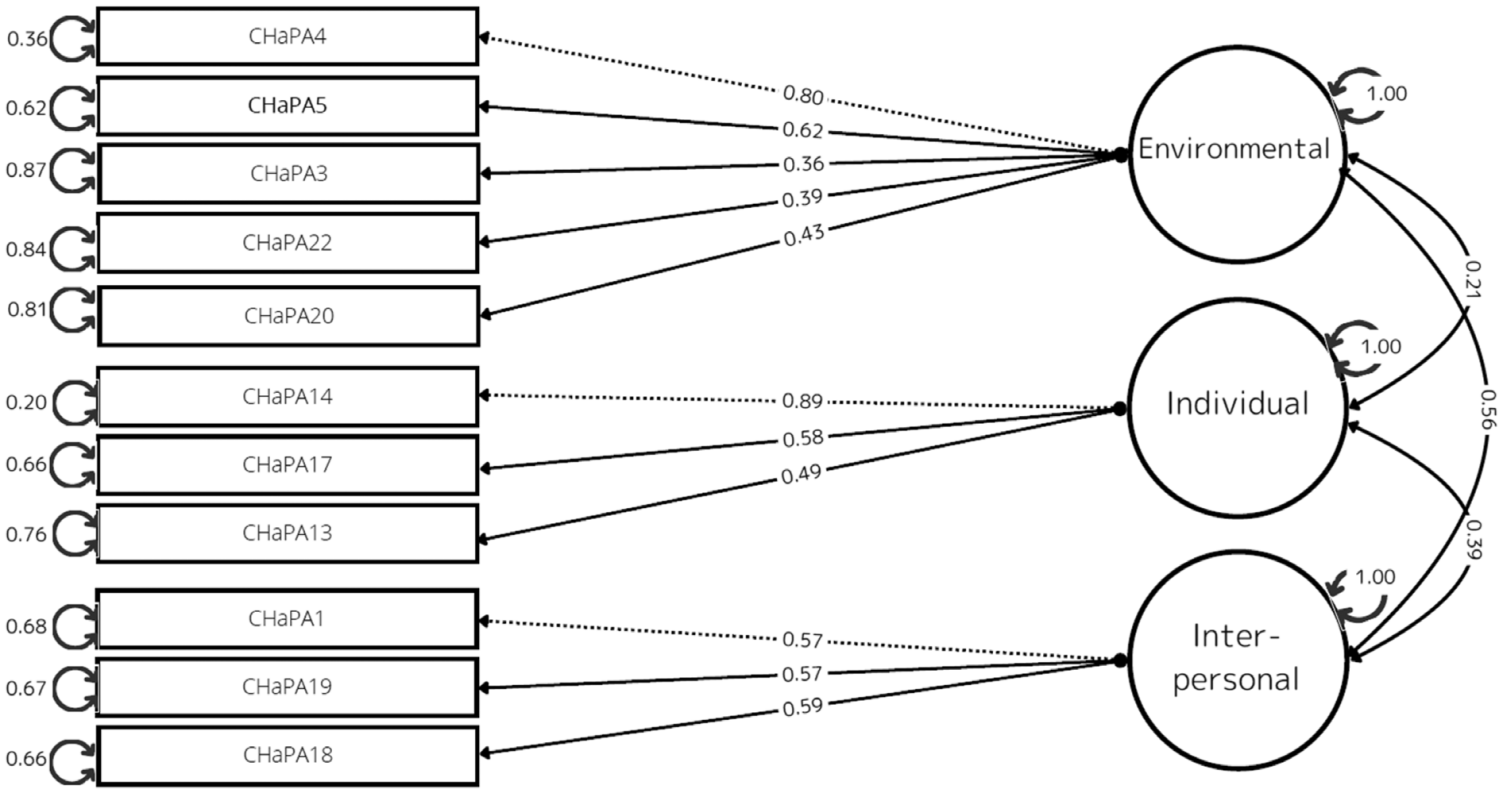
Path diagram of structural equation modelling. CHaPA, Checklist for Habitual Physical Activity

We repeated CFA to identify the combination of items for deletion based on factor loadings and structural interpretability, assuming a three‐factor structure. CFA on the CHaPA 17‐item version did not show sufficient goodness‐of‐fit index in the first analysis. Initially, Items 6, 7, and 11 were identified for removal due to their notably low factor loadings. Items 8, 12, and 15 exhibited factor loadings of 4.0 or higher, yet the model met the criteria for CFI, TLI, and RMSEA only upon their exclusion, along with other combinations. After considering the comprehensiveness of the constructs and model fits, Items 6, 7, 8, 11, 12, and 15 were ultimately excluded. Consequently, a final selection of 11 items was deemed optimal for the model fit. Table 4 shows the model fit for the three‐factor structure, with *χ* ^2^/df = 1.25 (*χ* ^2^ = 52.58, df = 41.00), CFI = 0.965, TLI = 0.952, and RMSEA = 0.038 of the CHaPA 11‐item version. Additionally, Figure 3 illustrates the path diagram of structural equation modelling for the CHaPA 11‐item version with the three‐factor structure, elucidating how the observed variables measure the latent variables corresponding to the constructs. The subscale correlations indicate there was a moderate positive correlation between individual and environmental factors (*r* = 0.56, *P* > 0.01), a weaker positive correlation between interpersonal and individual factors (*r* = 0.39, *P* > 0.01), and a relatively weaker positive correlation between environment and interpersonal factors (*r* = 0.21, *P* = 0.04). Table 5 shows the CHaPA 11‐item version created after the modification.

**Table 4:** CFT, TLI, RMSEA of the three‐factor structure

	*χ* ^2^/df	CFI	TLI	RMSEA
CHaPA 17‐item version	1.66	0.839	0.811	0.062
CHaPA 11‐item version	1.25	0.965	0.952	0.038

CFI, comparative fit index; CHaPA, Checklist for Habitual Physical Activity; TLI, Tucker‐Lewis index; RMSEA, root‐mean‐square error of approximations.

**Table 5:** The CHaPA 11‐item version

Factors	Items	Answer	
Utilising one's living area	Maintaining a habit of walking to the store	Yes	No
	Using units that are easy to understand (metres, minutes, steps) to keep track of activity level	Yes	No
	Tracking activity needed when travelling to neighbourhood landmarks (e.g., bus stops, supermarkets)	Yes	No
	Setting walking courses taking into account the local environment (e.g., crime prevention, safety, restrooms, resting places)	Yes	No
	Regularly utilising nearby stairs, slopes for physical activity	Yes	No
Integrating one's enjoyment, purpose, and roles	Getting daily enjoyment out of physical activity (e.g., meeting people, getting close to nature, observing the environment)	Yes	No
	Having a purpose other than exercise (e.g., looking after the community, visiting friends) for your physical activity	Yes	No
	Having a group role when doing physical activity with others (e.g., preparing and cleaning up, taking care of people and pets, managing equipment)	Yes	No
Planning regular physical activity and sharing it with others	Timeframe for physical activity is determined on a daily, weekly, or monthly basis	Yes	No
	Talking to family and friends about the physical activity you are doing	Yes	No
	Talking to your family doctor about the physical activity you are doing	Yes	No
	Total number of Yes		

### Criterion‐related validity

Table 6 shows Spearman's correlation coefficients between the CHaPA 11‐item version (total and subscale scores) and knowledge about national physical activity guidelines, Simple Frail Index, Social Frailty Index, SARC‐F, and PASE. The total score of the CHaPA 11‐item version exhibited a moderate negative correlation with the Social Frailty Index score (*ρ* = −0.42, *P* < 0.01). The total score of the CHaPA 11‐item version demonstrated a moderate positive correlation with the total score of PASE (*ρ* = 0.55, *P* < 0.01). Weak correlations were observed between the total score of the CHaPA 11‐item version and knowledge about national physical activity guidelines (*ρ* = 0.17, *P* > 0.05), the Simple Frail Index (*ρ* = 0.16, *P* > 0.05), and the SARC‐F (*ρ* = −0.12, *P* > 0.1).

**Table 6:** Criterion‐related validity of the CHaPA 11‐item version (*n* = 165)

Spearman's *ρ*					
	Knowledge about the national PA guideline	Simple Frail Index	Social Frailty Index	SARC‐F	PASE
CHaPA total	0.17*	−0.16*	−0.42**	−0.12	0.55**
Factor 1	0.11	−0.17*	−0.22*	−0.05	0.40**
Factor 2	0.13	−0.19*	−0.43**	0.05	0.49**
Factor 3	0.17*	−0.03	−0.20**	0.02	0.33**

* *P* > 0.05;

** *P* > 0.01.

CHaPA, Checklist for Habitual Physical Activity; PA, physical activity; PASE, Physical Activity Scale for the Elderly; SARC‐F, strength, assistance walking, rising from a chair, climbing stairs, and falls.

### Reliability

We excluded six additional datasets with missing values for the second CHaPA assessment, resulting in 165 entries for the test–retest and measurement error reliability assessment. Table 1 shows the characteristics of the participants (*n* = 165). The overall omega coefficient of the CHaPA 11‐item version was 0.90, indicating good internal consistency. The omega coefficients for each factor were also satisfactory, with Factor 1 = 0.86, Factor 2 = 0.81, and Factor 3 = 0.80. Table 7 presents the ICC of the CHaPA 11‐item version, demonstrating good test–retest reliability (ICC (1, 2) = 0.77, 95% CI = 0.34–0.89). We calculated the SEM to be 1.75.

**Table 7:** Intraclass correlation coefficient (ICC) of the Checklist for Habitual Physical Activity 11‐item version

	ICC (1, 2)	95% CI
Total	0.77	0.34–0.89
Factor 1	0.80	0.74–0.85
Factor 2	0.71	0.63–0.78
Factor 3	0.73	0.65–0.79

## DISCUSSION

We evaluated the CHaPA 22‐item version by item analysis, EFA and CFA, to reorganise and validate its conceptual framework and reliability. Initially, item analyses led to the removal of five items. Subsequent iterations of EFA and repeated CFA resulted in the removal of six additional items, leading to the establishment of the CHaPA 11‐item version with the three‐factor structure. The CHaPA 11‐item version encompassed three distinct factors: utilising one's living area resources, integrating one's enjoyment, purpose, and roles, and planning physical activity and sharing it with others. The final version demonstrated the optimal statistical and practical model fit. Moreover, the criterion‐related validity and reliability of the CHaPA 11‐item version affirmed its robustness as a checklist for evaluating behaviours that facilitate physical activity among older adults.

### Item analyses

The item analyses resulted in the removal of five items. Despite the endorsement frequency ranging from about 54% to 96%, we did not establish specific removal criteria based solely on the endorsement frequency. This decision aligned with recommendations suggesting that items should not be removed exclusively due to skewed response distributions.^26^ Regarding Item 2, although older adults require less sleep than when they were younger,^39^ sleep quality is essential for brain and body restoration, fatigue recovery, and enhancement of immune function.^40^ However, the impact of waking up at a consistent time on physical activity still needed to be verified, justifying Item 2 removal.

Both Items 9 and 21 pertain to behaviours regulating physical activity levels. While these behaviours may apply to older adults actively engaging in physical activities, the expressions could also be perceived as ways to intentionally lower physical activity levels. Consequently, these behaviours may be construed as mechanisms to curtail and inversely regulate active behaviour. Hence, these items did not align well with the I‐T correlation and G‐P analysis.

Regarding item 10, the phrase ‘the guidelines’ was deemed inappropriate, primarily due to the low awareness of national physical activity guidelines within the targeted population. A previous study, where we established content validity, highlighted low awareness of these guidelines.^13^ Despite listing the guidelines at the bottom of the checklist, this particular item was also deemed unsuitable for this population.

Concerning item 16, it was evident that older adults in their 90s have limited opportunities to engage in physical activity with peers older than themselves. Consequently, given the significant age group disparity observed, this item was deemed unsuitable as the item in a checklist applies to all older adults over 75, including those in their 90s.

### Construct validity

The results of the EFA supported a three‐factor structure: utilising one's living area resources, integrating one's enjoyment, purpose, and roles, and planning regular physical activity and sharing it with others without confounding or overlap. We adopted a three‐factor structure suggested in MAP and parallel analysis because the three‐factor structure aligned with the constructs when we originally developed the checklist.^13^, ^14^ Our original constructs for the CHaPA were based on the socio‐ecological model,^16^ categorising the factors related to physical activity into the individual, interpersonal, and environmental levels.^17^ We considered that integrating one's enjoyment, purpose, and role was at the individual level, planning regular physical activity and sharing it with others was at the interpersonal level, and using resources in the living area was at the environmental level. The restructured CHaPA's model was consistent with the social‐ecological model in that the daily behaviours that support habitual physical activity were constantly influenced and interacted at the individual, interpersonal, and environmental levels.^41^

The correct number of factors to retain in the model, resulting in a statistically meaningful yet comprehensible model, was one of the most critical decisions in EFA.^42^ We deleted Items 8, 12, 15 according to the model fit criteria. Even though knowledge of health benefits that Item 8 was expected to capture was considered a facilitator of physical activity among older adults,^43^ Item 8 did not fit the overall model. This item indicated perception rather than behaviour, exhibiting heterogeneous characteristics with other items that meant daily activities. The components of this checklist focused on specific behaviours and factors that promote physical activity. However, Items 12 and 15 relate not simply to the physical activity itself but to the interactions and individual experiences during that activity. These items relate to the social and emotional aspects and personal preferences during that activity rather than the specific behaviours that facilitate the physical activity. Therefore, they did not fit as components of the previous list.

CFA results indicated a satisfactory model fit. The correlation between the three factors explains the daily behaviours that promote physical activity. We recognised that the model derived by CFA was one of the models to interpret the data and that it was possible to assemble other models. While simplification of the checklist makes it easier to implement, one disadvantage is that users will no longer be informed of compensatory methods or ways to understand physical activity benefits, which were suggested within the deleted items. We recommend that interested researchers and practitioners refer to the underlying qualitative and content validation research articles for the deleted items.

### Criterion‐related validity

The CHaPA 11‐item version scores showed a moderate correlation^35^ with the PASE. In particular, Factor 2 substantially contributed more to the correlation, indicating an essential relationship between physical activity and personal enjoyment, purpose, and roles. The finding was in accordance with a previous systematic review on the importance of focusing on personal preferences in promoting physical activity among older adults.^44^ However, detailed associations between physical activity and having a purpose other than physical activity or finding a role during physical activity have yet to be clarified, and the present study provided important insights. The strong correlation between daily behaviours promoting physical activity and the amount of physical activity suggested that older adults could use the CHaPA 11‐item version to increase the amount of physical activity.

The CHaPA 11‐item version scores moderately correlated with the Social Frailty Index. The result was consistent with the report that people with greater social support for physical activity are more likely to engage in leisure time physical activity.^11^ Our hypothesis of a weak positive correlation between CHaPA and knowledge about physical activity guidelines was also supported.^19^ Although the hypothesis was that the CHaPA would demonstrate strong associations with the Simple Frail Index and SARC‐F, only weak associations were found. The result indicated that the CHaPA measures a construct different from the criteria for measuring loss of capacity or physical ageing.

### Reliability

The omega value of 0.90 indicates excellent reliability,^36^ suggesting the CHaPA 11‐item version measured the same construct when using this checklist. The test–retest reliability assessment confirmed that the CHaPA 11‐item version has good test–retest reliability. This scale is sensitive to meaningful changes because according to the SDC results, if scores on this scale change by more than five points, it can be interpreted as a change beyond measurement error.

### Study limitations and strength

We acknowledge several limitations. First, the sample population in this study had a higher proportion of women compared to the demographics of the Japanese population aged 75 and over.^45^ This discrepancy suggests that selection bias may have been present, potentially limiting the generalisability of the findings to the broader population in Japan. Additionally, we primarily included participants already engaged in physical activity, thus potentially excluding home‐bound or in‐patient individuals. However, the sample characteristics were deemed acceptable as Child (2006) stated that pooling data from diverse populations may obscure factors specific to each population.^46^ In this regard, we need to determine the validity and reliability of the CHaPA 11‐items version by limiting the number of community‐dwelling but home‐bound participants.

Even with these limitations, the CHaPA holds promise in bridging the gap between research and practice, indicating practical strategies based on older adults' behaviours that promote physical activity. Validation studies often focus on tools used by healthcare professionals, overlooking tools designed for self‐use by older adults to promote physical activity. For example, the PASE quantifies the amount of physical activity and focuses solely on exercise aspects. In contrast, the CHaPA allows respondents to learn about individual, interpersonal, and environmental‐focused behaviours that support physical activity as they complete the assessment. Respondents will know concrete beheviours to promote physical behaviours by using individual, interpersonal, and environmental perspectives, rather than solely exercise, to promote physical activity. A notable strength of the CHaPA was its item structure based on the PD approach, which allowed older adults to engage in these behaviours without needing special equipment, budgets, or facilities. Further research should be planned to explore the applicability of the CHaPA in diverse populations, such as older adults with mobility challenges and those confined to their homes, and to identify effective methodologies and appropriate timing for the broader application of the checklist.

## CONCLUSIONS

We examined the CHaPA 22‐item version and established the CHaPA 11‐item version as a valid and reliable instrument for assessing daily behaviours conducive to physical activity among individuals aged 75 years and older. However, further investigation is warranted to evaluate this checklist's dissemination methods and efficacy. Such examination is essential to ensure that the CHaPA 11‐item version can serve as a valuable resource for promoting healthy ageing and supporting older adults in maintaining an active lifestyle.

## AUTHOR CONTRIBUTIONS

Conceptualisation, K.A and Y. Ishibashi; methodology, K.A and Y. Ishibashi, T.T; formal analysis, K.A, Y. Ishibashi, and Y. Ikechi; investigation, K.A.; writing – original draft preparation, K.A; writing – review and editing, K.A, Y. Ishibashi, T.T, T. Ikechi, and H.I; supervision, Y. Ishibashi, H.I; funding acquisition, K.A. All authors have read and agreed to the published version of the manuscript.

## ETHICS STATEMENT

We obtained approval from the Ethics Committee of the Tokyo Metropolitan University (Approval No.23023).

## PATIENT CONSENT STATEMENT

We informed the candidates orally and in writing about the study methods, data confidentiality, voluntary participation, and the right to withdraw without any disadvantage. Study candidates became study participants upon submission of a consent form.

## Supporting information


**Table S1.** Inter‐item correlations of the Checklist for Habitual Physical Activity (CHaPA) 22‐item version.

## Data Availability

The data that support the findings of this study are available from the corresponding author upon reasonable request.
