# Supplementary material for: Validity and reliability of the Checklist for Habitual Physical Activity for people 75 years and older in Japan
Source: Psychogeriatrics. 2024 Sep 9;24(6):1282–96. doi: 10.1111/psyg.13189 (PMC12997146; doi:10.1111/psyg.13189)
Supplement: Supplementary file 1 [file PSYG-24-1282-s001.docx]

Supplemenrary Table 1 Inter-item correlations of CHaPA 22-item version

	CHaPA1	CHaPA2	CHaPA3	CHaPA4	CHaPA5	CHaPA6	CHaPA7	CHaPA8	CHaPA9	CHaPA10	CHaPA11	CHaPA12	CHaPA13	CHaPA14	CHaPA15	CHaPA16	CHaPA17	CHaPA18	CHaPA19	CHaPA20	CHaPA21	CHaPA22
CHaPA1	1	0.208662	0.077638	0.343471	0.231961	0.29803	0.096663	0.189153	-0.0528	-0.04694	0.163121	0.148104	0.008925	0.070259	0.398917	0.331567	0.118068	0.317885	0.346711	0.214819	0.063064	0.067884
CHaPA2	0.208662	1	0.066334	-0.0156	-0.04356	0.022954	0.062888	0.034919	-0.09071	0.059372	-0.0428	0.003109	-0.0401	-0.06789	0.040939	0.203679	0.078011	0.021582	0.062762	0.006484	-0.05624	0.009868
CHaPA3	0.077638	0.066334	1	0.276079	0.240649	0.112698	-0.0833	0.007784	0.012347	-0.02195	-0.00659	0.133399	0.083478	-0.06999	-0.07677	0.027608	0.027608	-0.00682	0.051	0.219365	0.03619	0.253181
CHaPA4	0.343471	-0.0156	0.276079	1	0.53224	0.145049	0.149712	0.299586	-0.06669	-0.0142	0.238525	0.249068	0.113389	0.151348	0.137071	0.172727	0.131579	0.209957	0.224438	0.332457	0.058521	0.266297
CHaPA5	0.231961	-0.04356	0.240649	0.53224	1	0.129639	0.014803	0.238558	0.013473	-0.02253	0.319948	0.14607	0.106277	0.113041	0.152595	0.068105	-0.01004	0.137421	0.171988	0.209499	-0.01646	0.187363
CHaPA6	0.29803	0.022954	0.112698	0.145049	0.129639	1	0.074692	0.120687	-0.06487	0.041191	0.304142	0.190957	0.03681	0.209532	0.097786	0.162944	0.145049	0.377475	0.24076	0.108673	0.001213	0.114671
CHaPA7	0.096663	0.062888	-0.0833	0.149712	0.014803	0.074692	1	0.155662	-0.0309	-0.0242	0.10458	-0.06081	0.089667	0.160572	-0.01669	0.085849	0.115163	0.177789	0.194648	0.009572	0.101361	0.047343
CHaPA8	0.189153	0.034919	0.007784	0.299586	0.238558	0.120687	0.155662	1	-0.06305	-0.01026	0.352039	0.261599	0.042629	0.130857	0.02243	0.044537	0.140981	0.104035	0.052635	0.194951	-0.0066	0.303717
CHaPA9	-0.0528	-0.09071	0.012347	-0.06669	0.013473	-0.06487	-0.0309	-0.06305	1	0.03175	0.019048	-0.06683	0.21974	0.067685	0.017514	0.081312	0.090227	-0.06628	0.111953	0.12266	-0.02094	-0.05955
CHaPA10	-0.04694	0.059372	-0.02195	-0.0142	-0.02253	0.041191	-0.0242	-0.01026	0.03175	1	0.079348	-0.01202	0.185689	0.086497	0.023911	0.146637	-0.0568	-0.06348	-0.1135	-0.12934	0.037558	0.157155
CHaPA11	0.163121	-0.0428	-0.00659	0.238525	0.319948	0.304142	0.10458	0.352039	0.019048	0.079348	1	0.298741	0.257583	0.338279	0.111763	0.238215	0.289637	0.31079	0.171339	0.162988	0.119246	0.196652
CHaPA12	0.148104	0.003109	0.133399	0.249068	0.14607	0.190957	-0.06081	0.261599	-0.06683	-0.01202	0.298741	1	0.12238	0.23984	0.019508	0.159856	0.224161	0.174106	0.02881	0.256694	-0.01341	0.244163
CHaPA13	0.008925	-0.0401	0.083478	0.113389	0.106277	0.03681	0.089667	0.042629	0.21974	0.185689	0.257583	0.12238	1	0.431892	0.085853	0.190357	0.264575	0.175517	0.130507	0.198538	0.097322	0.095618
CHaPA14	0.070259	-0.06789	-0.06999	0.151348	0.113041	0.209532	0.160572	0.130857	0.067685	0.086497	0.338279	0.23984	0.431892	1	0.249685	0.239233	0.520259	0.293232	0.176047	0.16636	0.097205	0.119651
CHaPA15	0.398917	0.040939	-0.07677	0.137071	0.152595	0.097786	-0.01669	0.02243	0.017514	0.023911	0.111763	0.019508	0.085853	0.249685	1	0.222873	0.25456	0.163	0.12407	0.077795	0.065088	-0.03096
CHaPA16	0.331567	0.203679	0.027608	0.172727	0.068105	0.162944	0.085849	0.044537	0.081312	0.146637	0.238215	0.159856	0.190357	0.239233	0.222873	1	0.2	0.290571	0.245941	0.219271	0.0615	-0.00575
CHaPA17	0.118068	0.078011	0.027608	0.131579	-0.01004	0.145049	0.115163	0.140981	0.090227	-0.0568	0.289637	0.224161	0.264575	0.520259	0.25456	0.2	1	0.172905	0.198542	0.182851	-0.0117	0.09154
CHaPA18	0.317885	0.021582	-0.00682	0.209957	0.137421	0.377475	0.177789	0.104035	-0.06628	-0.06348	0.31079	0.174106	0.175517	0.293232	0.163	0.290571	0.172905	1	0.333694	0.284133	0.236484	0.18356
CHaPA19	0.346711	0.062762	0.051	0.224438	0.171988	0.24076	0.194648	0.052635	0.111953	-0.1135	0.171339	0.02881	0.130507	0.176047	0.12407	0.24594	0.198542	0.333694	1	0.135744	0.071935	0.095541
CHaPA20	0.214819	0.006484	0.219365	0.332457	0.209499	0.108673	0.009572	0.194951	0.12266	-0.12934	0.162988	0.256694	0.198538	0.16636	0.077795	0.219271	0.182851	0.284133	0.135744	1	0.091247	0.231291
CHaPA21	0.063064	-0.05624	0.03619	0.058521	-0.01646	0.001213	0.101361	-0.0066	-0.02094	0.037558	0.119246	-0.01341	0.097322	0.097205	0.065088	0.0615	-0.0117	0.236484	0.071935	0.091247	1	-0.0037
CHaPA22	0.067884	0.009868	0.253181	0.266297	0.187363	0.114671	0.047343	0.303717	-0.05955	0.157155	0.196652	0.244163	0.095618	0.119651	-0.03096	-0.00575	0.09154	0.18356	0.095541	0.231291	-0.0037	1
